# Tracking the Biostimulatory Effect of Fractions from a Commercial Plant Protein Hydrolysate in Greenhouse-Grown Lettuce

**DOI:** 10.3390/antiox12010107

**Published:** 2022-12-31

**Authors:** Francesco Cristofano, Christophe El-Nakhel, Giuseppe Colla, Mariateresa Cardarelli, Youry Pii, Luigi Lucini, Youssef Rouphael

**Affiliations:** 1 Department of Agricultural Sciences, University of Naples Federico II, Via Università 100, 80055 Portici, Italy; 2 Department of Agriculture and Forest Sciences, University of Tuscia, 01100 Viterbo, Italy; 3 Faculty of Science and Technology, Free University of Bozen/Bolzano, 39100 Bolzano, Italy; 4 Department for Sustainable Food Process, Research Centre for Nutrigenomics and Proteomics, Catholic University of the Sacred Heart, 29122 Piacenza, Italy

**Keywords:** low nitrogen, ascorbic acid, fresh weight, produce quality, peptides, secondary metabolism, polyphenolics, chlorogenic acid, UHPLC, Orbitrap LC-MS/MS

## Abstract

Protein hydrolysate biostimulants are environmentally friendly options for the reduction of nitrogen input, but their plant growth-promoting mechanisms are still not completely unveiled. Here, to put the “signaling peptide theory” to the test, a greenhouse experiment was undertaken using low (1 mM) and optimal (8 mM) NO_3_-treated butterhead lettuce and three molecular fractions (PH1 (>10 kDa), PH2 (1–10 kDa) and PH3 (<10 kDa) fractions), in addition to the whole product Vegamin^®^: PH, in a randomized block design. PH1 and PH3 significantly increased fresh yield (+8%) under optimal (lighter leaves), but not under low (darker leaves) NO_3_ conditions. Total ascorbic acid, lutein and β-carotene increased with PH3, and disinapoylgentobiose and kaempferol-3-hydroxyferuloyl-sophorosie-7-glucoside content increased with PH (whole/fractions) treatments, particularly under low NO_3_ conditions. The complete hydrolysate and analyzed peptide fractions have differential biostimulatory effects, enhancing the growth and nutritional quality of lettuce.

## 1. Introduction

Nitrogen is a critical nutrient for plants, as plants rely on it for an insurmountable number of tasks, from nucleic acid building to enzymes and proteins. Furthermore, nitrogen is a critical element for photosynthesis, to which it provides the building blocks for chlorophyll and light-harvesting complexes [1]. Due to its importance at every level of plant physiology, farmers supply excessive amounts of this element to plants in the hope of obtaining better plant growth; however, this can increase nitrate concentrations in edible plant matter, the main dietary source for human consumption [2]. Moreover, by leaching into the soil, nitrate can also reach the water table, polluting drinking water sources [3].

One of the most cutting-edge solutions currently available for the reduction of nitrogen inputs is the use of plant biostimulants. As 2019′s EU regulation 1009 points out, plant biostimulants (PBs) “act in addition to fertilizers, with the aim of optimizing the efficiency of those fertilizers and reducing the nutrient application rates” [4]. Furthermore, PBs also pose themselves as a straightforward solution to increase plant functional quality parameters, which stem from the increase in secondary plant metabolites of known health-improving qualities, such as antioxidants and polyphenols [5]. Protein hydrolysate (PH) biostimulants are now a staple in the biostimulant scenario, and the literature shows that the use of such products can alleviate some of the yield losses due to deficient nitrogen supply and/or improve the nitrogen uptake efficiency in many greenhouse vegetable species such as lettuce, spinach and rocket [6,7,8]. Whilst a partial explanation of their effect may stem from the presence of amino acids, which are the building blocks for most plant tissues, research has postulated the role of the so-called signaling peptides to be essential in their effectiveness. Signaling peptides are short chains of amino acids (between 2 and 50) which induce hormone-similar responses at very low concentrations [9], and most of the PH literature point to the root hair-promoting peptide, a compound which has been found in widely available commercial formulations such as ‘Trainer’, as proof of this theory [10].

However, as plant source materials vary in their aminoacidic content and protein makeup, it is to be expected that biostimulants made from different protein sources may vary in the content of such peptides, and thus, effectiveness; research found this to be the case, as vegetal products from various botanical families exert distinct effects on either the growth or metabolism of plants, even when the same extraction process is performed [11]. One of the latest strategies to garner the best understanding of the inner workings of this biostimulant grouping has been molecular fractionation. Lucini and collaborators [9] found that the <1 kDa fractionation—around nine amino acid residues—of the PH ‘Trainer’ elicited the best root growth performance through IBA-like effects seen in the metabolomic data. This proof-of-concept work shows that the next generation of biostimulants can be assayed based on the potency of their single fractions and marketed accordingly.

On these bases, the aim of this work is to verify the influence of a newly developed PH biostimulant based on vegetal sources on the growth and plant phytochemical profile of lettuce plants in both optimal and low nitrogen conditions. To further prove the effectiveness of the low-molecular-weight peptides, the biostimulant was subjected to molecular fractionation in order to obtain the <1 kDa, 1–10 kDa and >10 kDa formulations. The experiment is meant to scale up previous lab work to greenhouse conditions, and to prove new biostimulant-making technologies for the industry, since the pressure on producers to find new and innovative products is stronger than ever, as nitrogen fertilizer is becoming both economically and environmentally unsustainable.

## 2. Materials and Methods

### 2.1. Growth Conditions, Experimental Design and Plant Material

A greenhouse experiment was carried out from 2 October 2020 (day after transplant 1, or DAT 1) to 12 November 2020 (DAT 42) in an unheated and passively ventilated greenhouse situated in the “Parco Gussone” area of the Department of Agricultural Sciences of the University of Naples “Federico II”, 40°48′ N, 14°20′ E, 29 m.s.l. Seedlings of *Lactuca sativa* L. cv. ‘Maravilla De Verano Canasta’, hereby defined as ‘Canasta’ (Pagano Domenico e Figli, Scafati, Salerno, Italy), a butterhead-type lettuce, were transplanted at the three-true leaves stage on 2 October 2020. Plants were transplanted into 1.6-L plastic pots containing growing substrate, which comprised of a mixture of 90:10 (*v*/*v*) 3 mm quartz sand (Vaga, Sabbie e Ghiaie Silicee, Località Sostegno—SP199 27010 Costa de’Nobili (PV) Italy) and perlite, respectively. The experimental setup consisted of four double rows with an inter- and intra-row distance of 35 and 20 cm, which represented a planting density of 14 plants m^−2^. A split-plot experimental design was employed, whereby the main factor consisted of the nutrient solution (NS) nitrogen dosage which was deemed either optimal (O) or low (L). The sub-factor consisted of four biostimulant (B) treatments and an untreated control which were arranged in a randomized complete block design with three replicates. In total, the design employed 30 experimental units, each consisting of five lettuce plants.

The base nutrient solution had the following composition: 1.5 mM phosphorus, 4 mM potassium, 2.5 mM sulfur, 1.25 mM magnesium, 1 mM sodium, 1 mM chloride, 20 μM iron, 9 μM manganese, 0.3 μM cupper, 1.6 μM zinc, 20 μM boron and 0.3 μM molybdenum. To this solution, two differential amounts of nitrogen (calcium nitrate) were added in order to provide for two nitrogen treatments: O, corresponding to 8 mM nitrate and 4mM calcium, and L, corresponding to 1 mM nitrate and 0.5 mM calcium. To ensure equal calcium concentration and guarantee iso-osmosis across NS treatments, the low nitrogen treatment was supplied with calcium chloride. The electrical conductivity (EC) of the resulting solutions was 1.6 ± 0.5. The pH of the solutions was monitored and kept at 5.8 ± 0.2 with a portable pH meter (HI 991301, Hanna Instruments Italia S.R.L., Ronchi di Villafranca Padovana (PD), Italy).

### 2.2. Biostimulant Characteristics

The commercially available protein hydrolysate Vegamin^®^ (Hello Nature Italia S.R.L., Rivoli Veronese (VR), Italy), hereby referred to as PH, made from vegetal sources, was the biostimulant chosen for this trial. Quantitative analysis of this biostimulant, obtained analogously to Sorrentino and collaborators [12], shows carbon and nitrogen contents of 25.6 and 17.1%, respectively. The aminogram of the product, expressed in g kg^−1^, was determined as: Ala (12), Arg (19), Asp (33), Cys (4), Glu (54), Gly (13), His (8), Ile (12), Leu (24), Lys (19), Met (4), Phe (16), Pro (15), Ser (17), Thr (11), Trp (4), Tyr (13) and Val (16).

The ferric-reducing antioxidant power (FRAP) and the total phenolic and flavonoid contents, measured analogously to Paul and collaborators [13], were as follows: 1.32 mM Fe^2+^ g^−1^, 8.94 mM gallic acid eq. g^−1^ and 770.3 µM quercitin eq. g^−1^. The elemental composition was determined as (g kg^−1^ biostimulant): N (50.0), P (0.9), K (41.1), Ca (10.9), Mg (0.5), Fe (0.024), Zn (0.010), Mn (0.001), B (0.005) and Cu (0.001).

Vegamin^®^ does not contain phytohormones as the analysis conducted by Sorrentino and collaborators [12] shows. PH fractionation and nitrogen content analysis were carried out according to the methodology employed by Lucini and collaborators [9]. The fractionation process consisted of two steps. First, the >10 kDa and <10 kDa fractions were obtained via the use of centrifuge filtering tubes (Amicon Ultra 15, Merck KGaA, Darmstadt, Germany). Second, after the use of 0.5–1 molecular cut-off (MWCO) cellulose acetate membranes (VWR, Milan, Italy), the <1 kDa and >1 kDa <10 kDa fractions were obtained. To sum up, biostimulants were separated in three fractions: <1 kDa, hereby called PH3, 1–10 kDa or PH2, and >10 kDa or PH1. Due to the use of water as the fractionation medium, dilution incurred and nitrogen contents of the obtained fractioned were subsequently determined as 0.11% (PH1), 0.16% (PH2) and 0.06% (PH3).

### 2.3. Biostimulant Treatments

Biostimulant treatments consisted of 4 levels of application of the Vegamin formulate and its fractions. The whole product, i.e., PH was applied at the manufacturer’s suggested rate of 3 mL biostimulant L^−1^ solution or 2.38 g biostimulant L^−1^. Due to dilution effects inherent to the fractionation process, the PH1, PH2 and PH3 treatment dosage rates were adjusted to provide plants with equal amounts of nitrogen to the unfractionated formulate; thus, dosage rates were 348.2 g L^−1^ for PH1, 251.74 g L^−1^ for PH2 and 659.0 g L^−1^ for PH3.

Treatments were administered to plants via foliar application using 10 L steel-bottle sprayers of the same model, which were tested for spraying volume accuracy using a graduated cylinder. Products were sprayed on lettuce plants until a uniform coverage was guaranteed, and polystyrene panels were used to avoid drift between different treatments. A total of five treatments were administered throughout the course of the trial, starting at DAT 13 and then every seven days.

### 2.4. Yield, Growth Assessment, Leaf Colorimetric Measurement and Sampling

At the end of the experiment (DAT 42), three plants per experimental unit were randomly selected for fresh weight measurements. Dry plant matter was obtained upon desiccation of the fresh matter using a forced-air drying oven at 60 °C until a constant weight was reached.

Colorimetric measurements were carried out using a Minolta CR-300 Croma Meter (Minolta Camera Co., Ltd., Osaka, Japan) which was calibrated prior to use against a standard white control. Leaf color was sampled on the adaxial side of six fully expanded leaves per experimental unit. Measurements are expressed in the CIELAB color space, comprising of L* (lightness), a* and b* (chromatic information). Visual color appearance of the plants was also validated using the CIEDE2000 indicator developed by the CIE Technical committee [14]. To provide more succinct colorimetric information, values were also converted to chroma (Chroma = ((a*)^2^ + (b*)^2^)^0.5^) and hue (Hue = ((Arctan (b*/a*)/2π) × 360) + 180).

The remaining two plants per experimental plot were chosen for the quality assays, and immediately transferred to a laboratory setting for further sampling. A set of fresh leaf samples was immediately used for the determination of leaf chlorophyl and total ascorbic acid contents. A further set of leaf samples was harvested and immediately transferred into liquid nitrogen. Samples were later stored at −80 °C for quality assays, and an aliquot was lyophilized using Martin Christ Alpha 1–4 freeze-drying equipment (Martin Christ Gefriertrocknungsanlagen GmbH, Osterode am Harz, Germany).

### 2.5. Carotenoids and Total Ascorbic Acid Determination

Chlorophyll Leaf pigment content was determined using 1 g of fresh leaf samples, which were extracted in pure acetone, kept in darkness for 15 min and subsequently centrifuged at 3000× *g* for five minutes. Pigment contents were determined using a Hach DR 2000 UV-Vis spectrophotometer (Hach Company, Loveland, CO, USA) by measuring their absorbance at 662 and 645 nm for chlorophyll a and b, respectively. Chlorophyll pigment quantification was made using the extinction coefficients found in Lichtenthaler and Buschmann’s work [15] and determined as mg 100 g^−1^ fresh weight (fw).

Leaf β-carotene and lutein analysis were carried out on 100 mg of lyophilized leaf matter. Extraction was first performed analogously to what is described by Kyriacou and collaborators [16]. In brief, sample material was firstly extracted in 6 mL of ethanol—0.1% butylated hydroxytoluene (BHT) mixture, then potassium hydroxide was added for saponification. Pigments were then extracted using hexane and later dried using nitrogen gas; 1 mL of chloroform was added to this residue and separated using Shimadzu Model LC 10 chromatography equipment (Shimadzu, Osaka, Japan) using a reverse phase 250 × 4.6 mm, 5 μm Gemini C18 column (Phenomenex, Torrance, CA, USA) as described by Kyriacou and collaborators [17]. Carotenoid contents were quantified as mg 100 g^−1^ fw.

The total ascorbic acid (TAA) assay was performed on fresh leaf tissue by way of the Kampfenkel [18] method, which determines the sum of ascorbic and dehydroascorbic acids by measuring sample absorbance at 525 nm against an ascorbic acid standard calibration curve. All measures were undertaken using the Hach DR 2000 UV-Vis spectrophotometer (Hach Company, Loveland, CO, USA) and expressed as mg AA 100 g^−1^ fw.

### 2.6. Leaf Mineral Analysis

All mineral content analyses were conducted on dried samples, which were processed using a model MF10.1 grinding mill (IKA-Werke GmbH & Co. KG, Staufen, Germany). Leaf mineral (NO_3_, P, K, Ca, S, Mg) and organic acid (citrate and malate) composition was determined using ICS-3000 ion chromatography equipment (Dionex, Sunnyvale, CA, USA). Anionic and cationic separations were obtained via the IonPac AS11-HC and IonPac CS12A analytical columns, and quantified against chromatography standards using electrical conductivity detectors (Dionex, Sunnyvale, CA, USA) as mentioned in detail by Rouphael and collaborators [19]. All mineral contents are quantified as mg 100 g^−1^ fw.

### 2.7. Leaf Polyphenolic Content

Polyphenol extraction was performed using 100 mg of freeze-dried leaf samples and 5 mL of a methanol/water (60:40 *v*/*v*) solution, according to Kyriacou and collaborators [16]. Briefly, qualitative and quantitative profiling of the compounds was also performed analogously to the previously mentioned paper using an Ultra High Pressure Liquid Chromatograph (UHPLC, Thermo Fisher Scientific, Waltham, MA, USA) which employed a 1.7 µm Biphenyl (100 × 2.1 mm) column (Phenomenex, Torrance, CA, USA). Later, mass spectrometry analysis was carried out using a Q Exactive Orbitrap LC-MS/MS (Thermo Fisher Scientific, Waltham, MA, USA). The targeted acquisition of polyphenolic compounds was carried out on parallel reaction monitoring (PRM) mode. This modality of acquisition allows a targeted MS/MS analysis using the mass inclusion list and expected retention times of the target analytes, with a 30 s time window, with the Orbitrap spectrometer operating in negative mode at 17,500 FWHM (*m*/*z* 200). The AGC target was set to 2e5, with the maximum injection time of 20 ms. The precursor ions in the inclusion list were filtered by the quadrupole at an isolation window of *m*/*z* 2 and fragmented in an HCD collision cell set at 30 Kv. Polyphenol quantification was done using calibration curves from authentical standards when available, and otherwise based on calibration curves of standard compounds belonging to the same chemical group and with a similar response. In particular, authentical standards were used for quantitative analysis of chlorogenic acid, ferulic acid, isorhamnetin-rutinoside, kaempferol diglucoside, quercetin glucoside and rutin, whereas semi-quantitative determination was carried out for coumaroyl-diglucoside (coumaric acid used as standard), disinapoylgentobiose (sinapic acid used as standard), synapoyl-hexose (sinapic acid used as standard) and for kaempferol 3-hydroxyferuloyl-sophorotrioside-7-glucoside (kaempferol-diglucoside used as standard). A mass tolerance of 5 ppm was employed. The instrument calibration was checked daily using a reference standard mixture obtained from Thermo Fisher Scientific.

Leaf polyphenolics contents were quantified as µg/g dw and then expressed as µg 100 g^−1^ fw based on the samples’ dry matter percentage.

### 2.8. Statistical Analysis, Cluster Analysis and Heatmap

Experimental data were subjected to bifactorial (nitrogen level × biostimulant) analysis of variance using the SPSS 28 software package (IBM, Armonk, NY, USA). Nitrogen dosage mean effect was compared by *t*-test. Biostimulants’ mean effects and factor interactions were separated by Tukey’s HSD test, performed at *p* ≤ 0.05.

A hierarchical cluster analysis (HC) on the quality and phytochemical composition of lettuce leaves was performed, and a heatmap was generated using the ClustVis online tool [20]. Matrix values were normalized as ln (x + 1), with Euclidean distance and complete linkage.

## 3. Results and Discussion

### 3.1. Lettuce Fresh Yield and Leaf Colorimetry Indices

**Figure 1:**
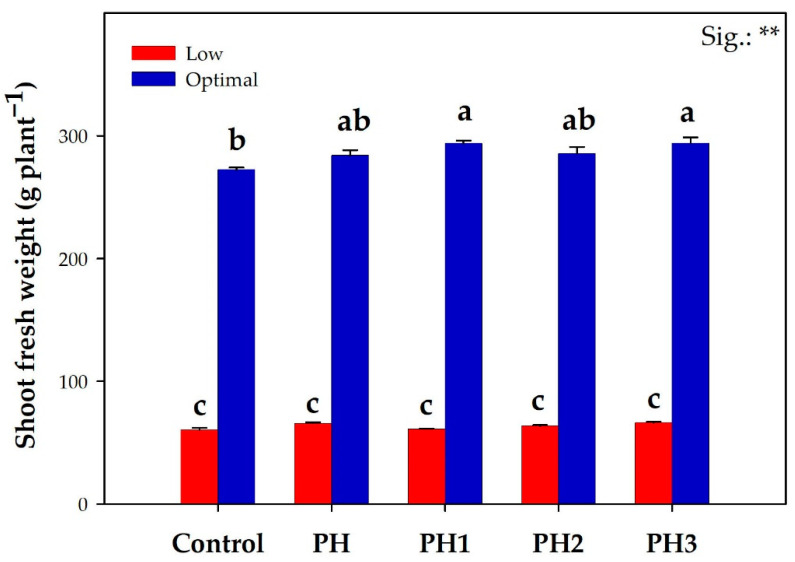
The commercial yield of lettuce plants as affected by nitrogen dosage and biostimulant application. All data are expressed as mean ± standard error, *n* = 3. Interaction data were deemed significant at *p* ≤ 0.01 (**). Different letters above the bars indicate significant differences according to Tukey’s HSD test, performed at *p* ≤ 0.05. PH: protein hydrolysate, molecular fractions PH1, PH2 and PH3 (>10 kDa, between 1 and 10 kDa, <10 kDa). Nitrogen dosage: Optimal = 8 mM NO_3_, Low = 1 mM NO_3_.

The results obtained by the greenhouse trial show two distinct outcomes in the case of the optimal and low nitrogen NS treatments (Figure 1; Supplementary Material Table S1). The commercial yield of lettuce was significantly—and greatly—affected by the nitrogen NS treatment, which decreased shoot fresh weights 4.5-fold in the low treatment.

Furthermore, significant biostimulant × nitrogen dosage effects were also recorded. In the optimal nitrogen group, both the H1 and H3 treatments were the best performing and recorded the highest shoot fresh weight, which translated into a 7.5% mean increase compared to their untreated control. Although PH and PH3 increased the shoot fresh weight by 9.0% compared to the control, the differences were not deemed significant in the suboptimal nitrogen group.

The marketable fresh weight increase in the optimal NS treatment is in line with the currently available literature on PH use in leafy vegetables, including lettuce, spinach and rocket, which see marketable yield increases after the formulates were applied [5,6,21,22]. However, this was the first research instance where the used treatments are molecular fractions deriving from the same biostimulant matrix, but also one where the widely available working theory behind them is not backed up by experimental data.

The current PH literature agrees upon the role of signaling peptides as one, if not the principal, driving factor behind plant growth in stress and non-stress conditions [23]. Evidence furthering this hypothesis comes from previous work by Lucini and collaborators [9], which showed that the PH3-equivalent fraction of the ‘Trainer’ PH biostimulant showed the best root growth when compared to both the other tested fractionates and an auxin hormone control. However, when analyzing the metabolic response to the PH3-equivalent action and the commercial formulate they found that, while, again, the former better fit an auxin-like footprint, the latter induced an accumulation of gibberellins and a down-accumulation of brassinosteroids, cytokinins and jasmonates [9].

Results also show that pitted against low nitrogen availability, the employed PH biostimulants could not ameliorate the overly deficient one-eighth of the optimal NS nitrogen conditions. This phenomenon is readily explained as nitrogen is critical to plant life as a constituent in both plant tissue and the photosynthetic machinery driving plant growth. C3 plants, which lettuce and many vegetable species are part of, allocate almost 24% of leaf nitrogen to thylakoids, and a large part of that nitrogen is employed for light-harvesting proteins [1]. Moreover, sustained nitrogen deficiency induces the breakdown of nucleic acids and enzymes, especially Rubisco, which irreversibly impairs photosynthesis and ultimately plant growth [24]. The conducted mineral analysis indisputably proves the point of an insufficient mineral amount to conduct basic plant metabolism, as nitrate, which plants use as nitrogen storage [25], was found to be depleted in plants from the low nitrogen NS group, which also explains the decrease in chlorophyll content found in this trial.

The significant shift in the color indices (Supplementary Material Table S2) is also a tell-tale sign of the low nitrogen stress. Average leaf color shows a CIE DE2000 of 6.80, which was noticeable by the naked eye. More in depth, compared to the O nitrogen treatment, L treated plants presented a darker leaf color (L*, −3.5%), of substantially higher redness (a*, +203.8%) and blueness (b*, −22.6%) coloration, in addition to lower color saturation (Chroma, −23.9%). Such a change in leaf color attributes indirectly reveals the production of anthocyanins, which has been previously described in the literature on red pigmented lettuce as a response to nitrogen deficiency stress, or nutrient solution deprivation, as shown for the same lettuce cultivar in research conducted by Ciriello and collaborators [26]. In particular, Becker and collaborators [27], consistently found an increase in anthocyanins in nitrogen-starved red lettuce, expressed as the cyanidin-derived cyanidin-3-O-(600-O-malonyl)-glucoside, a red pigmented molecule. Anthocyanins are desirable phytochemicals in vegetables, even when their low bioavailability is considered, as research shows in vivo and in vitro cardiovascular and cancer-preventing effects [28].

### 3.2. Leaf Mineral and Organic Acid Contents

The effects of the nitrogen dosage and biostimulant treatments are shown in Table 1. Nitrogen concentration effects were deemed significant across all studied leaf mineral parameters, whilst significant biostimulant effects were recorded in the case of phosphorous, potassium, sulfur, calcium and magnesium.

Such fresh weight mineral increases in the low nitrogen treatment probably stem from the concentration effect due to the elevated leaf dry matter recorded (data not shown). Leaf dry matter increases are compatible with the previous literature and could indicate an accumulation of carbon in the form of photosynthesis-derived starches, which, however, cannot be processed for amino acid assimilation due to the nitrogen-limiting conditions [29,30]. The highest increase in potassium contents may further this theory, as it serves as the regulatory ion for the transport of photosynthesis products and has been found to increase also in soybean leaves under severe nitrogen stress [31]. However, the most egregious result in the recorded reduction is, indeed, the leaf nitrate concentration, as the L nitrogen treatment showed nitrate levels that were almost 100-fold less than the O treatment. Whilst the two-magnitude order reduction in nitrate contents denoted in L treatment indeed proves the nitrogen treatment as being too low, it may represent a favorable outcome when considering that the majority of the daily human nitrate intake comes from vegetables [25]. Methemoglobinemia, a biochemical anemia which results from nitrate exposure, is largely reported as the most common nitrate-derived human illness, and excess nitrate and nitrite consumption has been further linked to neoplasiae, such as gastric cancer [32]. The consumption of nitrate-deficient vegetables may entail all the benefits of this food group, which include a proven reduction in the risk of chronic disease and premature mortality, whilst balancing the intake derived from other nitrate-rich sources such as cured meats and drinking water [32,33].

Nevertheless, it is also imperative to note that all recorded nitrate values in both optimal and low conditions were below the threshold imposed by the EU Regulation 1258/2011 for lettuce grown in protected culture.

Averaged across nitrogen treatments, phosphorous and potassium contents were significantly increased in comparison to the untreated controls by the PH3 treatment, which recorded 21.6% and 20.9% higher uptake. The PH1 treatment also recorded significantly higher leaf K (26.6%), S (21.9%) and Mg (28.2%) contents when compared to the untreated control.

Delving into the interaction data, the L*PH1 plants are characterized by significantly higher leaf calcium, compared to both O (+174.3%) and L (+59.4%) controls. Our results do not contrast the previous literature, which shows the potential of PH biostimulants to increase the use efficiency of supplied nutrients in greenhouse-grown leafy vegetables. Both Cristofano, Rouphael and their collaborators [5,34] described the effect as due to the ‘nutrient acquisition response’, which is the sum of increased carbon and nitrogen metabolism, root growth and gene expression for macronutrient transporters.

These results also prove that biostimulant supplementation to plants can improve their nutrient content, which can be especially useful to those populations exposed to nutrient deficiencies. The 2015–2020 dietary guides for Americans pit calcium and potassium as nutrients of public health concern due to under consumption by the populace [35].

However, the results show some pointers which can be addressed to discriminate PH1 and PH3.

First, the result obtained by the PH1 treatment in regards to calcium concentration shows a modulation of calcium influx to the shoot tissue. When all conditions are equal, shoot calcium concentration largely depends on element availability and transpirational water flux [36], which is impeded in nitrogen-limiting conditions as stomatal conductance decreases with decreasing nutrient supply [1].

In a second instance, both investigated organic acids were found to be differently modulated by the biostimulant treatments. When averaged across nitrogen NS concentration, the H3 treatment yielded a significantly higher concentration of malate, which increased by 24.7% compared to the untreated control. Citrate concentration was most affected by the H1 treatment, which determined an increase of 35.2% when compared to the control average. Similarly to the results obtained in this trial, previous literature has shown that malate and citrate were increased by the application of a different vegetal PH applied to lettuce [37]. Moreover, previous research has shown that PH biostimulants stimulate carbon metabolism, as gene expression relative to enzymes in the tricarboxylic acid (TCA) cycle was found to be upregulated after application [38,39]. However, such distinctive behavior between treatments underpins their dissimilarity, which could be due to the distinct modulation of the carbon metabolism cycle. Organic acids are crucial at the plant cell level, taking part in energy production, amino acid biosynthesis and adaptation to environmental changes, but most importantly contribute to human health due to their antioxidative role [40].

### 3.3. Leaf Pigments and Total Ascorbic Acid Content

Table 2 shows the effect of the biostimulant treatments on leaf pigments and total ascorbic acid content. Apart from leaf total ascorbic acid, the NS treatments induced significant differences in the studied parameters, while the biostimulant treatments influenced all the parameters, with β-carotene being influenced by NS x B interactions.

When averaged across nitrogen treatments, PH and PH3 manifested significant differences between each other (+34.9% in the former) regarding the amount of total chlorophylls. Significant leaf total ascorbic acid content variation was only found in respect of the biostimulant applications, and PH3 recorded the highest result with an increase of 41.5% when compared to the untreated control. Interaction data from the leaf β-carotene content shows the PH3 treatment recording the highest figures in both optimal (+76.4%, compared to the O*Control) and low (+51.9%, compared to the L*Control) nitrogen conditions. Lastly, when NS nitrogen treatments were averaged, lutein content was significantly increased (+35.5%) by the PH3 treatment when compared to the untreated control.

The collected data shows that across sustained nitrogen stress, plant response to biostimulant application revolves in part around the enzymatic and non-enzymatic antioxidant pathways, as highlighted by previous PH research showing similar outcomes [23]. Ascorbic acid or vitamin C, usually present in the anionic form ascorbate, is a key substrate of the ascorbate peroxidase-glutathione reductase (APX-GR) system, which serves to detoxify reactive oxidative species (ROS) in plant tissue, especially in the case of plant stress [41]. Plant carotenoids such as β-carotene and lutein serve both as enzymatic and non-enzymatic photooxidative protection by scavenging ROS, dissipating excess light energy via non photochemical quenching (NPQ, via the xanthophyll cycle [42]) and protecting cellular membranes in the case of stress [43]. However, it is particularly telling that the tested products induced distinct modulations of the studied phytochemicals, especially in the case of PH3. Lutein and β-carotene are the most abundant carotenoids in chloroplasts (70–75% of the total amount) [44], which, coupled with APX as the dominant chloroplast antioxidant system [45], show PH3 primed plants to work against photo-oxidative stress.

The enrichment of such phytochemicals is of interest in the context of human health improvement. While uncommon in developed countries, vitamin A deficiency is said by the WHO to be a public health problem in half of all countries [46]. Provitamin A carotenoids, which β-carotene is part of, are important to preserve eyesight; furthermore, while evidence on supplemental (i.e., exogenous) dietary carotenoids may be lacking, high intake from fruits and vegetables has proven health benefits, including a lower risk of developing chronic diseases, which confirms the importance of the plant matrix for nutrient absorption [43].

The same considerations can be made for vitamin C, as marginal, i.e., not scurvy-inducing, deficiency can occur in up to 15% of the general population, a figure which is doubled in the case of cigarette smokers, and can lead to a higher risk of all-cause mortality [47]. The data further the case for the application of products such as PH3 to induce the production of phytochemicals of interest; this can have a tangible and quick effect when compared to biotechnological practices, which could be used to achieve the same result, due to the complex regulatory network surrounding their accumulation [48].

### 3.4. Leaf Polyphenolics

Table 3 and Table 4 show the modulation of leaf polyphenolic contents by the NS and biostimulant treatments. When the biostimulant treatments are considered, the total leaf phenolic acid concentration was significantly affected (Table 3). In particular, when averaged across nitrogen treatments, the H2 treatment gave rise to the highest (+24.0%) significant increase in this parameter when compared to the untreated control.

When broken down into the analyzed components, chlorogenic acid gave the highest contribution (92.8% averaged across all treatments) to the total amount, and thus was similarly affected by the biostimulant applications. In fact, the H2 treatment still provided higher figures compared to the control, with an increase of 24.6% when considered across nitrogen treatments. Ferulic acid content was the second most present compound and was also affected by the tested biostimulants, as both H1 and H3 showed significant increases over the untreated counterparts by 33.1% on average.

When speaking about tissue flavonoid contents (Table 4), NS nitrogen dosage proved to be the most impactful factor when considering total content, as no significant difference was denoted from B treatment or N × B interaction. In fact, the L nitrogen treatment increased this parameter 6.7-fold compared to the optimal regimen. The most impactful driver of this change was the 7.3-fold increase in Quercetin-3-glucoside in the L treatment: this compound accounted for 92.5% of the L treatment’s flavonoid content. When the discrete compounds are considered, only kaempferol 3-hydroxyferuloyl-sophorotrioside-7-glucoside and isorhamnetin 3-rutinoside incurred in combinatory N × B interactions. In the former case, all biostimulants significantly increased leaf concentrations compared to their respective controls. However, both the L × H3 and L × PH proved to be the most effective by showing a 2.6- and a 4.5-fold increase compared to the optimal and low controls, respectively. When looking at the interaction data for isorhamnetin 3-rutinoside, it is shown that the H3 treatments in the L group were the most successful in augmenting this parameter, while in the O group, all the fractions were significantly effective when compared to their untreated control. Isorhamnetin-3-rutinoside was 2.3-fold higher in the L × H3 treatments versus the L control, and 2.1- and 3.3-fold compared to the O × H3 and O × Control treatments, respectively.

Polyphenolics are a class of molecules which stem from a common origin and serve as regulators of plant growth and as plant stress-response molecules. Starting from shikimate, they are the product of the differentiation of phenylalanine-derived cinnamate via the enzyme phenylalanine ammonia lyase (PAL), which starts the central phenylpropanoid pathway [49]. As the products of this pathway are extremely diverse, and contain polymers such as lignin and suberin and pigments such as anthocyanins, we have grouped them based on their structural similarity as phenolic acids, i.e., phenolic compounds with one carboxylic group, and flavonoids, i.e., compounds with a C6-C3-C6 ring structure [50,51]. However, irrespective of their structure, the evidence here obtained shows that under very low nitrogen conditions lettuce plants behave according to the hypotheses set out by Becker and collaborators, which translate into a shift in carbon metabolism due to a high C/N ratio, and nitrogen recycling via PAL, which leaves carbon skeletons for the phenylpropanoid synthesis [27]; this is particularly evident, as both the total phenolic and total flavonoid assays show marked increases due to the low nitrogen conditions. Chlorogenic acid is widely reported as the most present phenolic acid in lettuce [52] and biotechnological efforts to increase its concentration in plant tissues are documented in the literature due to its anti-carcinogenic and atherosclerosis-preventing activity [53]. Again, this shows how different molecular weight biostimulants impact plant metabolism in differential ways, as this phenolic acid has been shown to work as a connector of cell wall polymers, mechanically strengthening tissues as a barrier for pathogen stresses [54].

The recorded increases in leaf flavonoid contents are compatible with what is available in the current literature in lettuce grown in nitrogen-deficient media [27,52], and is a common response to stressful conditions. Becker et al. [27] found a general increase in the flavonoid contents of nitrogen-deprived lettuce plants, which is compatible with the results obtained in this trial. The production of kaempferol and quercetin-derived flavonoid molecules, which are key intermediates of anthocyanin production as they represent part of the biosynthetic pathway [50], was found to be highly induced by the nitrogen treatment. However, such an increase can prove interesting when considering that a diet rich in the compound is beneficial in many aspects of human health, from being antidiabetic to anti-inflammatory effects, and offering cardiovascular disease prevention [28]. However, the modulation of total phenolic acids and total flavonoids upon the different biostimulant treatments is partially in line with the results obtained by Giordano and collaborators [36], who applied a PH on two different cultivars of lettuce. In this previous work, total phenolic acids were significantly boosted in both cultivars, while total flavonoids were only significantly higher in one cultivar and steady in the second one, when subjected to PH treatment. The accumulation of antioxidant molecules, such as phenols and flavonoids, has been associated with the PH’s biostimulant modification of plant primary and secondary metabolism [5,13,37]. Indeed, according to the previous authors, plant-based PHs, as an action mode, trigger secondary metabolism via an increase in the expression of genes encoding phenylalanine, an ammonia-lyase enzyme. Anyhow, PH biostimulants have a proven track record of increasing nutrient use efficiency, plant stress tolerance and produce quality, all in accordance with EU regulation 1009/2019.

### 3.5. Cluster Analysis and Heatmap of the Accumulation of Phytochemicals

**Figure 2:**
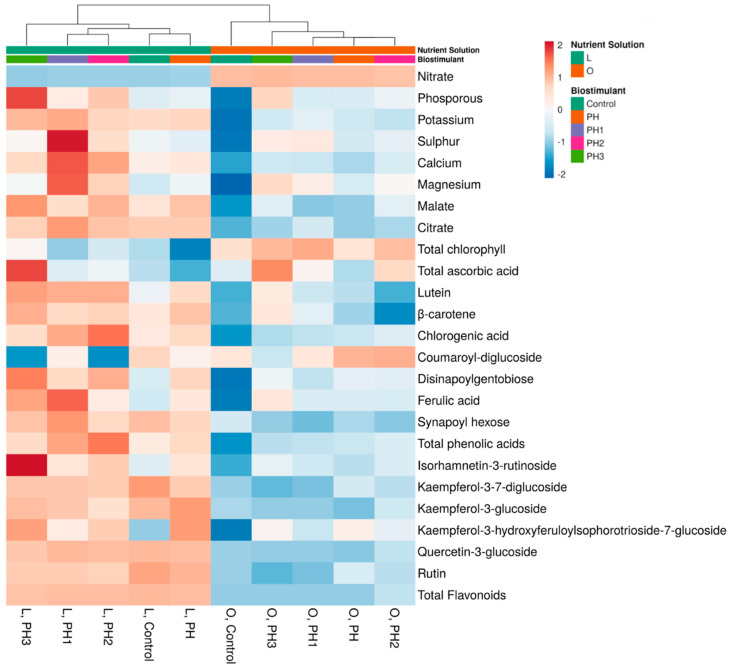
Cluster heat map analysis summarizing lettuce plants response to nitrogen dosage and biostimulant application. Original values are ln (x + 1)-transformed. Columns are clustered using Euclidean distance and complete linkage. PH: protein hydrolysate; molecular fractions PH1, PH2 and PH3 (>10 kDa, between 1 and 10 kDa, <10 kDa).

To provide a visual representation of the changes in phytochemical contents after the application of the biostimulant treatments, we have performed a hierarchical clustering analysis coupled with a heatmap, which can be seen in Figure 2.

The dendrogram presents two main clusters, which are divided based on the NS nitrogen treatment. In low nitrogen conditions, represented by the left cluster, the PH3 treatment is clearly separated from the other biostimulants and the control due to the increases in phosphorous, total ascorbic acid and isorhamnetin-3-rutinoside, as well as the lower coumaroyl-diglucoside content. The PH1 and PH2 clusters are associated with higher sulphur, calcium and magnesium, but also with an increase in total phenolic acids.

In optimal nitrogen conditions, we find two clusters represented by the control, which is separated by the biostimulant treatments. In this case, PH3 also separates from the remaining PH treatments due to the increases in phosphorous, total ascorbic acid, lutein, β-carotene and ferulic acid. PH, PH1 and PH2 treatments are clustered together and associated with coumaroyl-diglucoside, but also reduced lutein, β-carotene and ferulic acid contents.

## 4. Conclusions

The use of molecular fractionation is an adequate strategy to increase the potency of the PH-based products, and this trial represents a steppingstone in the lab-to-field journey. In optimal nitrogen conditions, both the PH1 and PH3 fractions successfully increased lettuce marketable yield by 7.9%, whereas in the low nitrogen conditions, biostimulant increases were not significant. However, across nitrogen conditions, we found that the best performing products also incremented the produce nutritional quality in ways that underline their different mode of action. PH3 induced a significant increase in total ascorbic acid (+41.5%), lutein (+35.5%) and β-carotene in both optimal (+76,4% compared to the O*Control) and low (+51.9%) conditions, which show that plants were primed to protect themselves from the oxidative stress by accumulating these compounds. The employed fractions also modulated the polyphenolic composition of the leaves in different, fraction-specific manners, as PH3 and PH1 induced a significantly higher accumulation of ferulic acid (+32.7%), when compared to total phenolic acid content, which was highest in the PH2 treatment (+24.6%). Again, whilst the limits of the study are found in a too low nitrogen concentration in the nitrogen stress group, it also successfully underlines the principle of PH biostimulants being a complex matter, as PH1 and PH3 resulted in similar growing prowess, but modified plant secondary metabolites in a distinct way. However, the qualitative results here recorded also provide a practical use case of the fractions to improve the functional quality of produce.

## Figures and Tables

**Table 1:** Mineral and organic acids analysis of lettuce plants as affected by nitrogen dosage and biostimulant application.

Source of Variance	NO_3_	P	K	S	Ca	Mg	Malate	Citrate
	(mg kg^−1^ fw)	(mg 100 g^−1^ fw)	(mg 100 g^−1^ fw)	(mg 100 g^−1^ fw)	(mg 100 g^−1^ fw)	(mg 100 g^−1^ fw)	(mg 100 g^−1^ fw)	(mg 100 g^−1^ fw)
Nutrient Solution (NS)								
Optimal N (O)	1060 ± 46	20.5 ± 0.5	264 ± 11	3.51 ± 0.12	33.6 ± 1.4	18.7 ± 0.6	192 ± 6	19.6 ± 1.1
Low N (L)	11.3 ± 0.6	22.1 ± 0.6	407 ± 9	3.87 ± 0.15	57.7 ± 3.1	20.1 ± 0.5	283 ± 7	60.0 ± 2.4
*t*-test	***	*	***	*	***	*	***	***
Biostimulant (B)								
Control	534 ± 242	19.3 ± 0.9 ^b^	291 ± 44 ^b^	3.28 ± 0.23 ^b^	37.0 ± 4.7 ^c^	16.8 ± 0.7 ^c^	211 ± 25 ^c^	35.9 ± 9.3 ^b^
PH	543 ± 243	20.6 ± 0.8 ^b^	336 ± 30 ^ab^	3.5 ± 0.19 ^ab^	41.0 ± 4.1 ^bc^	18.7 ± 0.4 ^bc^	234 ± 22 ^abc^	37.3 ± 8.6 ^b^
PH1	526 ± 233	21.0 ± 0.3 ^ab^	368 ± 32 ^a^	4.2 ± 0.21 ^a^	54.9 ± 9.1 ^a^	21.5 ± 0.9 ^a^	225 ± 21 ^bc^	48.6 ± 10.8 ^a^
PH2	505 ± 229	21.9 ± 0.8 ^ab^	332 ± 30 ^ab^	3.74 ± 0.17 ^ab^	50.0 ± 6.5 ^ab^	20.2 ± 0.8 ^ab^	255 ± 17 ^ab^	40.0 ± 9.7 ^ab^
PH3	568 ± 254	23.5 ± 0.5 ^a^	352 ± 32 ^a^	3.73 ± 0.16 ^ab^	45.3 ± 5.0 ^abc^	20.0 ± 0.5 ^ab^	263 ± 23 ^a^	37.2 ± 8.1 ^b^
	n.s.	**	**	*	**	***	**	*
NS × B								
O × Control	1058 ± 139	18.1 ± 1.0	194 ± 15	2.96 ± 0.28	27.2 ± 3.1 ^e^	15.4 ± 0.5	161 ± 9	15.4 ± 1.1
O × PH	1074 ± 121	20.4 ± 1.2	275 ± 11	3.44 ± 0.05	32.7 ± 1.0 ^de^	18.3 ± 0.4	184 ± 1	18.4 ± 1.1
O × PH1	1041 ± 77	20.4 ± 0.4	301 ± 17	3.82 ± 0.26	35.1 ± 3.4 ^de^	19.9 ± 1.2	183 ± 17	24.8 ± 3.4
O × PH2	999 ± 139	20.9 ± 0.6	269 ± 15	3.56 ± 0.18	37.0 ± 3.0 ^cde^	19.4 ± 1.	216 ± 6	20.1 ± 1.1
O × PH3	1127 ± 106	22.5 ± 0.6	280 ± 10	3.77 ± 0.21	35.8 ± 1.9 ^de^	20.7 ± 0.6	213 ± 1	19.3 ± 0.5
L × Control	10.7 ± 2.0	20.5 ± 1.3	387 ± 15	3.61 ± 0.27	46.8 ± 1.5 ^bcd^	18.1 ± 0.3	260 ± 25	56.4 ± 3.2
L × PH	12.9 ± 1.8	20.8 ± 1.2	397 ± 27	3.56 ± 0.43	49.4 ± 3.7 ^bcd^	19.1 ± 0.8	284 ± 7	56.3 ± 2.5
L × PH1	11.4 ± 0.6	21.6 ± 0.1	435 ± 19	4.57 ± 0.14	74.6 ± 3.6 ^a^	23.1 ± 0.5	267 ± 15	72.4 ± 1.5
L × PH2	11.4 ± 1.9	22.8 ± 1.4	394 ± 18	3.92 ± 0.27	62.9 ± 5.6 ^ab^	21.0 ± 1.1	293 ± 5	59.9 ± 8.8
L × PH3	10.0 ± 1.0	24.6 ± 0.2	423 ± 9	3.69 ± 0.29	54.7 ± 5.5 ^bc^	19.3 ± 0.6	313 ± 11	55.2 ± 1.4
	n.s.	n.s.	n.s.	n.s.	*	n.s.	n.s.	n.s.

All data are expressed as mean ± standard error, *n* = 3. n.s., *, **, ***: non-significant or significant at *p* ≤ 0.05, 0.01 and 0.001, respectively. Nitrogen dosage means (O = 8 mM NO_3_, L = 1 mM NO_3_) were compared by *t*-test. Different letters within each column indicate significant differences according to Tukey’s HSD (*p* = 0.05). PH: protein hydrolysate; molecular fractions PH1, PH2 and PH3 (>10 kDa, between 1 and 10 kDa, <10 kDa).

**Table 2:** Chlorophyll, auxiliary pigments, total ascorbic acid content of lettuce plants as affected by nitrogen dosage and biostimulant application.

Source of Variance	Total Chlorophylls	TAA	β-Carotene	Lutein
	(mg 100 g^−1^ fw)	(mg AA 100 g^−1^ fw)	(mg 100 g^−1^ fw)	(mg 100 g^−1^ fw)
Nutrient Solution (NS)				
Optimal N (O)	95.0 ± 3.4	139 ± 6.00	1.54 ± 0.11	1.54 ± 0.1
Low N (L)	58.5 ± 3.4	132 ± 7.43	2.47 ± 0.12	2.30 ± 0.06
*t*-test	***	n.s.	***	***
Biostimulant (B)				
Control	71.9 ± 7.0 ^ab^	121 ± 6 ^bc^	1.53 ± 0.16 ^c^	1.69 ± 0.18 ^b^
PH	64.8 ± 11.0 ^b^	112 ± 3 ^c^	1.95 ± 0.24 ^bc^	1.91 ± 0.23 ^ab^
PH1	79.0 ± 11.7 ^ab^	131 ± 6 ^bc^	2.12 ± 0.25 ^ab^	1.99 ± 0.13 ^ab^
PH2	80.7 ± 8.8 ^ab^	141 ± 10 ^b^	1.96 ± 0.35 ^b^	1.72 ± 0.28 ^b^
PH3	87.5 ± 6.8 ^a^	172 ± 5 ^a^	2.48 ± 0.18 ^a^	2.29 ± 0.15 ^a^
	*	***	***	**
NS × B				
H × Control	86.9 ± 2.6	126 ± 13	1.23 ± 0.08 ^e^	1.30 ± 0.01
H × PH	86.6 ± 9.8	117 ± 7	1.51 ± 0.08 ^de^	1.45 ± 0.05
H × PH1	103 ± 8.2	136 ± 11	1.57 ± 0.09 ^cde^	1.72 ± 0.12
H × PH2	98.8 ± 7.0	149 ± 8	1.23 ± 0.17 ^e^	1.14 ± 0.08
H × PH3	99.4 ± 7.2	166 ± 11	2.17 ± 0.24 ^abcd^	2.10 ± 0.25
L × Control	56.9 ± 3.4	117 ± 0	1.83 ± 0.17 ^bcde^	2.09 ± 0.08
L × PH	43.1 ± 6.5	107 ± 2	2.39 ± 0.28 ^abc^	2.38 ± 0.20
L × PH1	54.7 ± 5.4	125 ± 7	2.67 ± 0.07 ^ab^	2.26 ± 0.05
L × PH2	62.5 ± 2.8	133 ± 20	2.7 ± 0.24 ^ab^	2.30 ± 0.22
L × PH3	75.6 ± 6.0	177 ± 2	2.78 ± 0.15 ^a^	2.48 ± 0.09
	n.s.	n.s.	*	n.s.

All data are expressed as mean ± standard error, *n* = 3. n.s., *, **, ***: non-significant or significant at *p* ≤ 0.05, 0.01 and 0.001, respectively. Nitrogen dosage means (O = 8 mM NO_3_, L = 1 mM NO_3_) were compared by *t*-test. Different letters within each column indicate significant differences according to Tukey’s HSD (*p* = 0.05). PH: protein hydrolysate; molecular fractions PH1, PH2 and PH3 (>10 kDa, between 1 and 10 kDa, <10 kDa. TAA: total ascorbic acid.

**Table 3:** Phenolic acids profile of lettuce plants as affected by nitrogen dosage and biostimulant application.

Source of Variance	Chlorogenic Acid	Coumaroyl-Diglucoside	Disinapoylgentobiose	Ferulic Acid	Synapoyl-Hexose	Total Phenolic Acids
	(µg 100 g^−1^ fw)	(µg 100 g^−1^ fw)	(µg 100 g^−1^ fw)	(µg 100 g^−1^ fw)	(µg 100 g^−1^ fw)	(µg 100 g^−1^ fw)
Nutrient Solution (NS)						
Optimal N (O)	9736 ± 245	13.1 ± 0.8	3.17 ± 0.12	753 ± 32	32.1 ± 1.3	10537 ± 255
Low N (L)	13431 ± 416	11.4 ± 1.0	4.26 ± 0.15	899 ± 39	80.3 ± 4.8	14426 ± 436
*t*-test	**	**	***	n.s.	***	**
Biostimulant (B)						
Control	10308 ± 907 ^b^	13.8 ± 2.0	2.83 ± 0.21 ^d^	685 ± 57 ^b^	60.0 ± 11.1	11069 ± 955 ^b^
PH	11545 ± 936 ^ab^	13.4 ± 1.3	3.85 ± 0.19 ^bc^	813 ± 37 ^ab^	53.7 ± 11.6	12429 ± 980 ^ab^
PH1	11990 ± 1046 ^ab^	12.9 ± 1.6	3.68 ± 0.26 ^c^	903 ± 81 ^a^	61.1 ± 14.6	12971 ± 1123 ^ab^
PH2	12847 ± 1043 ^a^	11.6 ± 1.4	3.98 ± 0.27 ^b^	809 ± 55 ^ab^	50.8 ± 10.4	13722 ± 1070 ^a^
PH3	11227 ± 680 ^ab^	9.59 ± 0.6	4.25 ± 0.33 ^a^	919 ± 46 ^a^	55.5 ± 12.5	12216 ± 718 ^ab^
	**	n.s.	***	*	n.s.	**
NS × B						
O × Control	8450 ± 403	13.7 ± 3.9	2.38 ± 0.09 ^d^	610 ± 23	38.5 ± 2.5	9114 ± 418
O × PH	10017 ± 428	14.4 ± 1.1	3.43 ± 0.05 ^c^	759 ± 15	32.9 ± 2.6	10827 ± 446
O × PH1	9959 ± 736	12.8 ± 1.2	3.12 ± 0.12 ^c^	761 ± 44	28.5 ± 1.9	10765 ± 690
O × PH2	10521 ± 130	14.4 ± 0.4	3.40 ± 0.13 ^c^	765 ± 100	30.2 ± 3.3	11335 ± 71
O × PH3	9732 ± 32	10.4 ± 0.8	3.54 ± 0.12 ^c^	869 ± 75.	30.7 ± 1.8	10645 ± 106
L × Control	12166 ± 709	13.8 ± 1.9	3.28 ± 0.02 ^c^	760 ± 100	81.5 ± 12.2	13024 ± 754
L × PH	13073 ± 1364	12.5 ± 2.5	4.27 ± 0.06 ^b^	867 ± 61	74.6 ± 15.3	14031 ± 142
L × PH1	14022 ± 893	13.0 ± 3.3	4.23 ± 0.06 ^b^	1045 ± 104	93.8 ± 0.9	15178 ± 980
L × PH2	15173 ± 118	8.74 ± 1.1	4.56 ± 0.05 ^ab^	852 ± 56	71.4 ± 10.5	16109 ± 143
L × PH3	12722 ± 278	8.87 ± 0.5	4.96 ± 0.15 ^a^	970 ± 46	80.4 ± 12.7	13786 ± 322
	n.s.	n.s.	*	n.s.	n.s.	n.s.

All data are expressed as mean ± standard error, *n* = 3. n.s., *, **, ***: non-significant or significant at *p* ≤ 0.05, 0.01 and 0.001, respectively. Nitrogen dosage means (O = 8 mM NO_3_, L = 1 mM NO_3_) were compared by *t*-test. Different letters within each column indicate significant differences according to Tukey’s HSD (*p* = 0.05). PH: protein hydrolysate; molecular fractions PH1, PH2 and PH3 (>10 kDa, between 1 and 10 kDa, <10 kDa).

**Table 4:** Flavonoids profile of lettuce plants as affected by nitrogen dosage and biostimulant application.

Source of Variance	Isorhamnetin 3-Rutinoside	Kaempferol 3,7-Diglucoside	Kaempferol 3-Glucoside	Kaempferol 3-Hydroxyferuloyl-Sophorotrioside-7-Glucoside	Quercetin 3-Glucoside	Rutin	Total Flavonoids
	(µg 100 g^−1^ fw)	(µg 100 g^−1^ fw)	(µg 100 g^−1^ fw)	(µg 100 g^−1^ fw)	(µg 100 g^−1^ fw)	(µg 100 g^−1^ fw)	(µg 100 g^−1^ fw)
Nutrient Solution (NS)							
Optimal N (O)	3.33 ± 0.12	0.89 ± 0.11	3.82 ± 0.30	1.55 ± 0.10	67.8 ± 4.5	1.78 ± 0.22	80.0 ± 4.8
Low N (L)	5.50 ± 0.42	6.59 ± 0.78	12.6 ± 0.70	1.81 ± 0.14	495 ± 15	13.8 ± 1.52	535 ± 16
*t*-test	***	***	***	***	***	***	***
Biostimulant (B)							
Control	3.12 ± 0.26 ^d^	5.11 ± 2.58	8.60 ± 2.16	0.69 ± 0.09 ^d^	295 ± 98	10.2 ± 5.16	323 ± 107
PH	4.08 ± 0.41 ^c^	3.60 ± 1.08	9.25 ± 2.72	1.92 ± 0.17 ^a^	279 ± 97	8.69 ± 2.70	307 ± 103
PH1	4.17 ± 0.36 ^bc^	3.23 ± 1.25	7.75 ± 2.02	1.32 ± 0.13 ^c^	294 ± 102	6.46 ± 2.50	316 ± 109
PH2	4.58 ± 0.47 ^b^	3.41 ± 1.09	7.32 ± 1.32	1.63 ± 0.17 ^b^	291 ± 90	6.82 ± 2.18	315 ± 95
PH3	6.11 ± 0.99 ^a^	3.85 ± 1.28	8.25 ± 2.29	1.89 ± 0.18 ^a^	268 ± 89	7.69 ± 2.55	294 ± 96
	***	n.s.	n.s.	***	n.s.	n.s.	n.s.
NS × B							
O × Control	2.56 ± 0.09 ^d^	0.73 ± 0.10	4.06 ± 1.07	0.51 ± 0.03 ^g^	77.1 ± 18.6	1.46 ± 0.20	86.5 ± 20.0
O × PH	3.18 ± 0.09 ^cd^	1.40 ± 0.07	3.32 ± 0.78	1.55 ± 0.07 ^c^	67.8 ± 7.6	2.79 ± 0.15	80.0 ± 8.2
O × PH1	3.41 ± 0.09 ^c^	0.52 ± 0.12	3.50 ± 0.02	1.03 ± 0.06 ^ef^	71.9 ± 5.0	1.05 ± 0.23	81.4 ± 4.9
O × PH2	3.59 ± 0.07 ^c^	1.02 ± 0.31	4.62 ± 0.63	1.26 ± 0.02 ^de^	91.5 ± 8.2	2.04 ± 0.63	104 ± 9.7
O × PH3	3.90 ± 0.04 ^c^	0.74 ± 0.28	3.59 ± 0.60	1.49 ± 0.01 ^cd^	72.0 ± 5.7	1.49 ± 0.57	82.5 ± 6.2
L × Control	3.68 ± 0.06 ^cd^	9.48 ± 3.76	13.1 ± 1.22	0.88 ± 0.06 ^f^	513 ± 19.8	19.0 ± 7.52	559 ± 30
L × PH	4.97 ± 0.11 ^b^	5.80 ± 0.98	15.2 ± 1.10	2.30 ± 0.03 ^a^	491 ± 42.7	14.6 ± 1.28	534 ± 45
L × PH1	4.93 ± 0.22 ^b^	5.94 ± 0.70	12.0 ± 1.52	1.60 ± 0.07 ^c^	515 ± 58.2	11.9 ± 1.41	552 ± 62
L × PH2	5.56 ± 0.34 ^b^	5.79 ± 0.36	10.0 ± 1.02	1.99 ± 0.06 ^b^	491 ± 20.5	11.6 ± 0.72	526 ± 22
L × PH3	8.33 ± 0.09 ^a^	5.91 ± 0.22	12.9 ± 2.06	2.28 ± 0.03 ^a^	464 ± 30.6	11.8 ± 0.45	506 ± 32
	***	n.s.	n.s.	**	n.s.	n.s.	n.s.

All data are expressed as mean ± standard error, *n* = 3. n.s., **, ***: non-significant or significant at *p* ≤ 0.01 and 0.001, respectively. Nitrogen dosage means (O = 8 mM NO_3_, L = 1 mM NO_3_) were compared by *t*-test. Different letters within each column indicate significant differences according to Tukey’s HSD (*p* = 0.05). PH: protein hydrolysate; molecular fractions PH1, PH2 and PH3 (>10 kDa, between 1 and 10 kDa, <10 kDa).

## Data Availability

The datasets generated for this study are available on request to the corresponding author.

## Supplementary Materials

The following supporting information can be downloaded at: https://www.mdpi.com/article/10.3390/antiox12010107/s1, Table S1: Shoot fresh weight of lettuce plants as affected by nitrogen dosage and biostimulant application; Table S2: Colorimetric measurements of lettuce plants as affected by nitrogen dosage and biostimulant application.
