# Supplementary material for: Tracking the Biostimulatory Effect of Fractions from a Commercial Plant Protein Hydrolysate in Greenhouse-Grown Lettuce
Source: Antioxidants (Basel). 2022 Dec 31;12(1):107. doi: 10.3390/antiox12010107 (PMC9854572; doi:10.3390/antiox12010107)
Supplement: Supplementary file 1 [file antioxidants-12-00107-s001.zip › antioxidants-2130635-supplementary.pdf]

Table S1: Shoot fresh weight of lettuce plants as affected by nitrogen dosage and biostimulant application

Source of variance	Shoot fresh weight (g $plant^{-1}$)
Nutrient Solution (NS)	
Optimal N (O)	286 ± 3
Low N (L)	63.5 ± 0.7
t-test	***
Biostimulant (B)	
Control	166 ± 47 b
PH	175 ± 49 ab
PH1	177 ± 52 a
PH2	175 ± 50 ab
PH3	180 ± 51 a
	**
NS × B	
O×Control	272 ± 2 b
O×PH	284 ± 4 ab
O×PH1	294 ± 2 a
O×PH2	286 ± 5 ab
O×PH3	294 ± 5 a
L×Control	60.6 ± 1.4 c
L×PH	65.8 ± 0.7 c
L×PH1	61.3 ± 0.1 c
L×PH2	63.7 ± 0.5 c
L×PH3	66.3 ± 0.6 c
	**

All data are expressed as mean ± standard error, n = 3. **, *** significant at $p \leq 0.01$ and 0.001, respectively. Nitrogen dosage means (O = 8mM NO3, L = 1mM NO3) were compared by *t*-Test. Different letters within each column indicate significant differences according to Tukey's HSD (p = 0.05). PH: Protein Hydrolysate, molecular fractions PH1, PH2 and PH3 (>10 kDa, between 1 and 10 kDa, <10 kDa)

Table S2: Colorimetric measurements of lettuce plants as affected by nitrogen dosage and biostimulant application

Source of variance	L*	a*	b*	Chroma	Hue angle
**Nutrient Solution (NS)**					
Optimal N (O)	43.7 ± 0.4	-7.05 ± 0.43	26.6 ± 0.4	27.7 ± 0.4	107 ± 2
Low N (L)	42.2 ± 0.4	-2.32 ± 0.59	20.6 ± 0.9	21.1 ± 0.9	133 ± 10
t-test	**	***	***	***	*
**Biostimulant (B)**					
Control	42.3 ± 1.2	-3.62 ± 1.68	22.7 ± 2.1	23.4 ± 2.3	139 ± 18
PH	42.5 ± 0.4	-4.57 ± 1.44	22.1 ± 2.3	22.8 ± 2.5	118 ± 9
PH1	42.8 ± 0.7	-5.20 ± 0.93	24.9 ± 1.0	25.6 ± 1.1	119 ± 11
PH2	44.1 ± 0.5	-5.09 ± 0.97	24.7 ± 1.1	25.4 ± 1.2	107 ± 6
PH3	42.9 ± 0.6	-4.93 ± 1.52	23.9 ± 1.5	24.7 ± 1.6	118 ± 15
	n.s.	n.s.	n.s.	n.s.	n.s.
NS × B					
O×Control	44.3 ± 0.7	-6.21 ± 1.76	26.6 ± 1.9	27.5 ± 2.0	115 ± 9
O×PH	42.2 ± 0.5	-7.19 ± 0.85	25.7 ± 0.7	26.8 ± 0.9	105 ± 1
O×PH1	44.0 ± 0.6	-6.94 ± 1.01	26.9 ± 0.8	27.9 ± 0.8	104 ± 2
O×PH2	44.5 ± 0.9	-7.14 ± 0.66	27.0 ± 0.4	28.0 ± 0.4	105 ± 1
O×PH3	43.5 ± 1.0	-7.75 ± 0.68	27.0 ± 0.6	28.2 ± 0.6	106 ± 1
L×Control	40.3 ± 1.5	-1.02 ± 2.05	18.7 ± 1.9	19.2 ± 1.9	162 ± 30
L×*PH	42.9 ± 0.5	-1.96 ± 1.67	18.4 ± 3.6	18.8 ± 3.7	130 ± 16
L×PH1	41.6 ± 0.8	-3.45 ± 0.49	22.8 ± 0.6	23.3 ± 0.6	133 ± 20
L×PH2	43.7 ± 0.4	-3.04 ± 0.35	22.4 ± 0.5	22.8 ± 0.6	109 ± 12
L×PH3	42.2 ± 0.3	-2.12 ± 1.76	20.8 ± 0.9	21.2 ± 0.9	131 ± 32
	n.s.	n.s.	n.s.	n.s.	n.s.

All data are expressed as mean ± standard error, n = 3. ns, *, **, *** non-significant or significant at $p \leq 0.05$, 0.01 and 0.001, respectively. Nitrogen dosage means (O = 8mM NO3, L = 1mM NO3) were compared by *t*-Test. Chroma = $((a^*)^2 + (b^*)^2)^{0.5}$), Hue = ((Arctan (b*/a*)/2π)*360)+180). Different letters within each column indicate significant differences according to Tukey's HSD ($p = 0.05$). PH: Protein Hydrolysate, molecular fractions PH1, PH2 and PH3 (>10 kDa, between 1 and 10 kDa, <10 kDa)
